# β-Hydroxybutyrate Suppresses Lipid Accumulation in Aged Liver through GPR109A-mediated Signaling

**DOI:** 10.14336/AD.2019.0926

**Published:** 2020-07-23

**Authors:** A Kyoung Lee, Dae Hyun Kim, EunJin Bang, Yeon Ja Choi, Hae Young Chung

**Affiliations:** ^1^Department of Pharmacy, College of Pharmacy, Pusan National University, Busan 46241, Korea; ^2^Department of Biopharmaceutical Engineering, Division of Chemistry and Biotechnology, Dongguk University, Gyeongju 38066, Korea

**Keywords:** β-hydroxybutyrate, GPR109A, AMPK, ER stress, lipid accumulation, aged liver

## Abstract

Dietary interventions such as prolonged calorie restriction (CR) and intermittent fasting provide health benefits including a reduction in the inflammatory burden and regulation of energy metabolism. During CR, β-hydroxybutyrate (BHB) level is elevated in the serum. BHB is a ligand of GPR109A, which inhibits lipolysis and exerts anti-inflammatory effects on cells. During aging, comorbidities related to dyslipidemia are significantly associated with fatty liver. However, the underlying mechanisms of BHB in hepatic ER stress and dyslipidemia are unclear and remain to be elucidated. Here, we used aged rats that were administered with BHB and compared the modulatory effects of BHB through the GPR109A/AMPK pathway on the hepatic endoplasmic reticulum (ER) stress and lipid accumulation to CR rats. BHB caused suppression of hepatic ER stress and lipid accumulation through GPR109A/AMPK pathway in the aged rats. Aged rats of both treatment groups showed reduced cAMP level and PKA phosphorylation. Furthermore, AMPK-Ser173 phosphorylation via PKA was decreased, whereas AMPK-Thr172 phosphorylation was increased by BHB and CR. Further supporting evidence was provided in HepG2 cells that BHB inhibited ER stress and lipid accumulation induced by palmitate. These results suggest that BHB activates GPR109A and regulates the activation of AMPK. These findings were further confirmed by GPR109A-siRNA transfection *in vitro*. In addition, BHB treatment elevated the protein levels of AMPK leading to significant inhibition of hepatic steatosis, whereas AMPK-siRNA treatment abolished these effects. Taken together, these findings suggest that BHB could be a effective molecule that mimics CR in ameliorating age-related hepatic lipid accumulation via GPR109A signaling pathway.

Dyslipidemia is a pathological condition commonly characterized by increased serum triglycerides (TGs) and low-density lipoprotein (LDL) levels along with decreased high-density lipoprotein (HDL) cholesterol level [1]. These abnormalities of lipid metabolism are influenced by dysfunctions of the liver in maintaining metabolic homeostasis [2]. Hepatic lipid accumulation is representative of the pathological phenomenon of age-related liver diseases such as hepatitis, cirrhosis, and cancer [3]. Endoplasmic reticulum (ER) stress disrupts lipid metabolism and induces hepatic lipotoxicity [4]. ER stress is defined as a disturbance of ER homeostasis that leads to an impaired protein synthesis process leading to accumulation of unfolded and misfolded proteins in the ER lumen that triggers an ER stress response or unfolded protein response (UPR) signaling pathway. ER stress has been associated with the occurrence of age-related metabolic disorders such as diabetes mellitus and other pathological conditions, for example, dysregulated hepatic lipid metabolism [5, 6]. In order to respond and cope with ER stress, UPR signaling transduction is activated by three major transmembrane proteins located in the ER, i.e., ER-localized protein sensors, namely, inositol-requiring enzyme 1 (IRE1), protein kinase RNA-like endoplasmic reticulum kinase (PERK), and activating transcription factor 6α (ATF6α) [3]. ER stress increases the TG level and enhances the expression of SREBP-1c, FAS, and ACC leading to lipid accumulation in the normal hepatic and hepatoma cells [7]. However, the molecular mechanisms of how CR and β-hydroxybutyrate (BHB) delay aging-related liver ER stress need to be further investigated. Understanding these mechanisms is of great importance as this could lead to the identification of new therapeutic targets for age-associated ER stress and hepatic lipid metabolism.

In human and non-human primates, CR has beneficial effects on prolonging human life and reduces the incidence of age-related diseases, such as obesity, diabetes mellitus, hypertension, cardiovascular diseases, and cancers [8]. The level of one of the ketone bodies, BHB, is increased post dietary interventions, such as starvation and CR [9, 10]. However, the molecular mechanism of how CR can delay aging-related liver lipid accumulation needs to be further investigated. Understanding the mechanisms underlying CR is of great importance as this could lead to the identification of new therapeutic targets for age-associated liver diseases. Ketone bodies are small lipid-derived molecules that serve as a circulating source of energy for tissues during fasting or prolonged exercise [11]. Ketone bodies refer to three molecules, BHB, acetoacetate, and acetone. Most of the ketone body production occurs in the liver although smaller amounts may be produced in other tissues through aberrant expression of the ketogenic enzymes or reversal of the ketolysis pathway [12]. Ketone bodies mediate the neuroprotective effects of CR and also mimic the effect of CR in extending the lifespan of *C. elegans* [13]. Some studies have shown that ketone bodies circulating in low concentrations exert anti-inflammatory effects. Furthermore, BHB suppresses inflammasome formation through reduction of ER stress via AMPK activation [10]. GPR109A is demonstrated to be a receptor of BHB, and its activation leads to anti-lipolysis [14]. However, although many studies have suggested the molecular mechanisms of BHB and its anti-lipogenesis effects, their relationship with anti-lipogenic signaling remains unknown.

GPR109A is a seven-transmembrane G protein-coupled receptor (GPR) belonging to the Gαi family that is expressed in the adipocytes of white and brown adipose tissues, keratinocytes, immune cells, and the epithelial cells of the colon and retina. A recent study showed that GPR109A is expressed in the normal human and mouse livers [15]. BHB binds to GPR109A and leads to the dissociation of the heterotrimeric G protein complex into Gαi and Gβγ subunits. The exchange of GTP from GDP results in the activation of Gαi, thereby inhibiting the activity of adenylyl cyclase (AC) [16]. Therefore, cAMP-dependent protein kinase, PKA, was suppressed by a reduced cAMP level. PKA phosphorylates the AMPK inactivation site, AMPK Ser-173 [17].

Increased phosphorylation of AMPK-Ser173 reciprocally decreases AMPK-Thr172, which is consistent with the down-regulation of AMPK activity [18]. AMPK activation prevents ER stress response and lipid accumulation [19]. Furthermore, a previous study showed that BHB activates AMPK in rats [10]. AMPK stimulates glucose uptake and FA oxidation in the liver, whereas it inhibits gluconeogenesis and synthesis of cholesterol, FA, and protein [20].

Aging represents the collective changes in all organisms over time, which encompasses physical, psychological, and social changes. Age-related diseases include diabetes, cancer, arthritis, dementia, vascular diseases, obesity, and metabolic syndrome [21]. Insulin resistance is a pathological condition in which the cells fail to respond to normal insulin signals to store glucose within the tissues. As a result, hyperglycemia and hyperinsulinemia occur because of reduced glucose uptake from the tissues in response to insulin. There is a consequent increase in insulin secretion by the pancreatic beta cells in an attempt to maintain glucose homeostasis. In addition, patients with diabetes overproduce glucose and TGs contributing to hyperglycemia and hypertriglyceridemia [22]. However, the molecular mechanisms by which BHB regulates aging-related ER stress and hepatic steatosis need to be further investigated.

## MATERIALS AND METHODS

### Materials

The chemical reagents were obtained from Sigma (St. Louis, MO, USA). The antibodies were obtained from Santa Cruz Biotechnology (Santa Cruz, CA, USA) or Abcam (Cambridge, MA, USA). Polyvinylidene difluoride (PVDF) membranes were obtained from Millipore Corporation (Bedford, MA, USA). Dokdo-MARK^TM^ protein size marker was obtained from ElpisBiotech (Daejeon, Korea). (±)-Sodium 3-hydroxybutyrate was purchased from Sigma-Aldrich (St. Louis, MO, USA). All other materials obtained were of the highest available grade. The PKA inhibitor was obtained from Cayman Chemical (MI, USA).

### Animal experiments

Pathogen-free male Sprague Dawley rats were obtained from Samtako (Osan, Korea) and were fed a standard laboratory diet (Superfeed Co., Wonju, Kangwon, Korea) *ad libitum*. The animals at 6 and 24 months of age were used as young and old rats, respectively. Briefly, the male rats were fed a diet comprising 21% soybean protein, 15% sucrose, 43.65% dextrin, 10% corn oil, 0.15% α-methionine, 0.2% choline chloride, 5% salt mix, 2% vitamin mix, and 3% Solka-Floc. The *ad libitum* (AL)-fed group had free access to food and water. The CR animals were fed 60% of the food intake of their AL-fed littermates beginning at 30 days of age. There were 4 rats in each experimental group. To estimate the effects of BHB on lipid accumulation, BHB was administered to the old rats by oral gavage (10 and 100 mg/kg/day) for 30 days. All animal studies were approved by the Institutional Animal Care Committee of Pusan National University (Approval Number PNU-2013-0437). This experiment followed the guidelines on animal experiments issued by the Pusan National University.

### Biochemical analysis

Blood samples were collected after the animals of each group were killed. Kits were used to measure the concentration of the metabolites in serum as follows: TG (Shinyang Co, Korea) and free fatty acids (FFAs). Serum FFA level was determined using the FFA assay kit SICDIA NEFAZYME (Shinyang Co, Korea). BHB concentrations were determined with a BHB detection kit (Stanbio Laboratory, USA).

### Cell culture

Human hepatoma cells line, HepG2 cells, were obtained from the American Type Culture Collection (ATCC, Manassas, VA, USA). The cells were grown in Dulbecco's Modified Eagle Medium (DMEM, Nissui, Tokyo, Japan) containing 2 mM L-glutamine, 100 mg/ml streptomycin, 2.5 mg/L amphotericin B, and 10% heat-inactivated fetal bovine serum (FBS). The cells were maintained at 37 ºC in a humidified atmosphere containing 95% air/5% CO_2_. The cells were discarded after 3 months and then new cells were obtained from the frozen stock. The cells in the exponential phase were used for all experiments.

### FFA preparation

Palmitate (100 mM, Sigma-Aldrich) stock was prepared in 0.1 M NaOH at 70 °C and filtered. FFA-free BSA [5% (w/v), Sigma-Aldrich] solution was prepared in double-distilled H_2_O and filtered. 5 mM FFA/5% BSA solution was prepared by complexing FFA with 5% BSA in a 60 °C water bath.

### Oil Red-O staining

Oil Red O staining is used to identify cellular neutral lipid droplet accumulation. The cells were stained by the Oil Red O method. After treatments, the cells were washed three times with ice-cold PBS and fixed with 4% formalin for 30 min, and then dipped in 60% isopropanol for 5 min. After fixing, the cells were washed and stained with Oil Red O solution for 60 min at room temperature. After staining, the cells were washed three times with PBS to remove the unbound stain.

### Measurement of tissue and cellular TG

The liver tissues and cells were homogenized in phosphate-buffered saline (PBS). TG was extracted with methanol: chloroform (1:2) at room temperature for 2 h. After removing the impurities using a filter, the TG liquid solvent was dried. The TG level was estimated using a kit (Bioassay Systems, Hayward, CA, USA).

### Measurement of intracellular cAMP concentration

The intracellular cAMP was extracted from the cells and the concentration was assayed with a microplate reader (Bio-Rad, Hercules, CA, USA) using a cAMP assay kit (R&D Systems, Minneapolis, MN, USA) according to the manufacturer’s instructions. A commercial cAMP ELISA kit (Cell Biolabs) was used to determine the total cAMP level following the manufacturer’s instructions. The tissue and cell lysates were used to calculate cAMP values. Protein from cell lysates was calculated using bicinchoninic acid (BCA) protein assay kit (Thermo Scientific). The cAMP concentration was normalized to that of the protein.

### Protein isolation from tissues and cells

One gram of liver tissue was homogenized with 1 ml of homogenate buffer containing 100 mM Tris (pH 7.4), 20 mM β-glycerophosphate, 20 mM NaF, 2 mM Na_3_VO_4_, 1 mM EDTA, 0.5 mM PMSF, 1 μM pepstatin, and 80 mg/L trypsin inhibitor. The solution was centrifuged at 900 *g* at 4 °C for 15 min. The supernatant was re-centrifuged at 12,000 *g* at 4°C for 15 min to obtain the cytosol fraction.

The cells were washed with PBS, and then 1 ml of ice-cold PBS was added. The pellets were harvested by centrifuging at 1,000 *g* at 4°C for 5 min. The pellets were suspended in ProEX CETi lysis buffer solution (Translab, Daejeon, Korea), and was used for extraction of total protein from the cells according to the manufacturer’s instructions.

### Protein analysis and western blotting

The protein concentration was determined by BCA method (Thermo Fisher Scientific, Rockford, IL) using BSA as a standard. Homogenized liver tissues and cell lysed samples were boiled for 5 min with a gel-loading buffer [pH, 6.8; composed of 125 mM Tris-HCl, 4% sodium dodecyl sulfate (SDS), 10% 2-mercaptoethanol and 0.2% bromophenol blue] in a ratio of 1:1. An equal amount of protein was separated by sodium dodecyl sulfate-polyacrylamide gel (SDS-PAGE) using 6-17% gels. The gels were subsequently transferred onto Immobilon-P transfer membrane (Millipore Corp, Bedford, MA, USA). The membrane was immediately placed in a blocking solution [5% non-fat dry milk in TBS-Tween (TBS-T) buffer containing 10 mM Tris, 100 mM NaCl, and 0.1% Tween 20; pH 7.5] at room temperature for 1 h. The membrane was washed in TBS-T buffer for 30 min and incubated with primary antibody at room temperature for 2 h. After 30 min-washing in TBS-T buffer, the membrane was incubated with a secondary antibody at room temperature for 1 h. After 40 min-washing in TBS-T buffer, antibody labeling was detected using ECL following the manufacturer’s instruction and exposed to a radiographic film. Pre-stained blue protein markers were used for the determination of the molecular weight.

**Table 1:** Primer sequences for qPCR.

Gene	Forward (5’-3’)	Reverse (3’-5’)
**Rat**		
**ACOX1**	TCAGCAGGAGAAATGGATGC	TGGAAGTTTTCCCAAGTCCC
**CPT1**	AAGCTGTGGCCTTCCAGTTC	GGATGAAATCACACCCACCA
**PPARγ**	CCAGGTGACCCTCCTCAAGT	CTGCAGCAGGTTGTCTTGGA
**FAS**	AGTGAGTGTACGGGAGGGCT	GCTGGGACACATGTGATGGT
**PPARα**	AGGAAGCCATTCTGCGACAT	CTCTGCAGGTGGAGCTTGAG
**SCD**	GTTCTCTGAGACACACGCCG	GGATGAAGCACATGAGCAGG
**ACC**	GGCACTCTGATCTGGTCACG	GCTCCGCACAGATTCTTCAA
**Human**		
**PPARα**	GGAAAGCCCACTCTGCCCCCT	AGTCACCGAGGAGGGGCTCGA
**SREBP-1c**	CGACATCGAAGACATGCTTCAG	GGAAGGCTTCAAGAGAGGAGC
**PPARγ**	CATTCTGGCCCACCAACTTTGG	TGGAGATGCAGGCTCCACTTTG
**FAS**	GACATCGTCCATTCGTTTGTG	CGGATCACCTTCTTGAGCTCC
**SCD**	GTTCTCTGAGACACACGCCG	GGATGAAGCACATGAGCAGG
**CPT1**	GGTCCAGGTAGAGCTCAGGC	GTGCTCTGAGGCCTTTGTCA
**ACOX1**	AGGTCACAGCTGTCCAACCA	TTACCCAGCCCTGGCTTAAT

### Gene expression analysis with real-time PCR

Tissue RNA was purified using RiboEx Total RNA (GeneAll, Seoul, Korea) according to the manufacturer’s instruction. Total RNA (2 μg) was resolved in RNase-free water and was reverse-transcribed with a cDNA synthesis kit (Geneall, Seoul, Korea). Quantitative Real-Time PCR was performed using a SensiFAST SYBT NO-ROS kit (BIOLINE, London, UK) and a CFX Connect System (Bio-Rad Laboratories, Inc., Hercules, CA, USA). The primer sequences are shown in Table 1.

**Table 2:** Changes in body weight, food intake and liver weight intake during the experimental period.

	Young	Old	BHB 10	BHB 100	CR
**Body Weight**					
**Initial (g)**	609.5±23.1	756.3±10.0	758.3±14.6	752.0±13.4	752.0±18.2
**Final (g)**	631.8±14.4	789.0±14.2	699.3±23.9	706.5±13.6	699.5±15.4
**Food Intake**					
**Initial (g)**	21.2±1.4	22.7±0.7	18.1±1.1	19.3±0.7	13.1
**Final (g)**	23.0±0.9	25.3±1.5	20.5±0.9	20.8±1.6	15.1
**Liver weight (g)**	20.5±0.77	29.9±1.56^###^	25.1±0.57^*^	23.9±0.20^**^	23.4±0.32^**^
**Liver weight/Body weight(g/g)**	0.032±0.0012	0.038±0.0020^#^	0.036±0.0008	0.034±0.0003^*^	0.033±0.0005^*^

Data are expressed as mean ± SEM. Significance:

# p < 0.05,

### p < 0.001 *vs.* Young (6 months);

* p < 0.05,

** p < 0.01 *vs.* Old (24 months)

### Small interfering RNA-mediated gene silencing

To knock down GPR109A and AMPK in HepG2 cells, we utilized scrambled or GPR109A-siRNAs (IDT, Coralville, Iowa, USA) and AMPK-siRNA (Santa Cruz, CA, USA) obtained from a commercial source. Transfection was carried out using the Lipofectamine 2000 reagent (Invitrogen, Grand Island, New York, USA). The cells were treated with GPR109A-siRNA (20 nM) or AMPK-siRNA (20 nM) Lipofectamine complexes in Opti-MEM (Invitrogen) without serum. After incubation for 4 h, the transfection medium was replaced with fresh medium, and the cells were incubated for another 44 h, during which they were treated with BHB at the indicated times.

**Figure 1.**
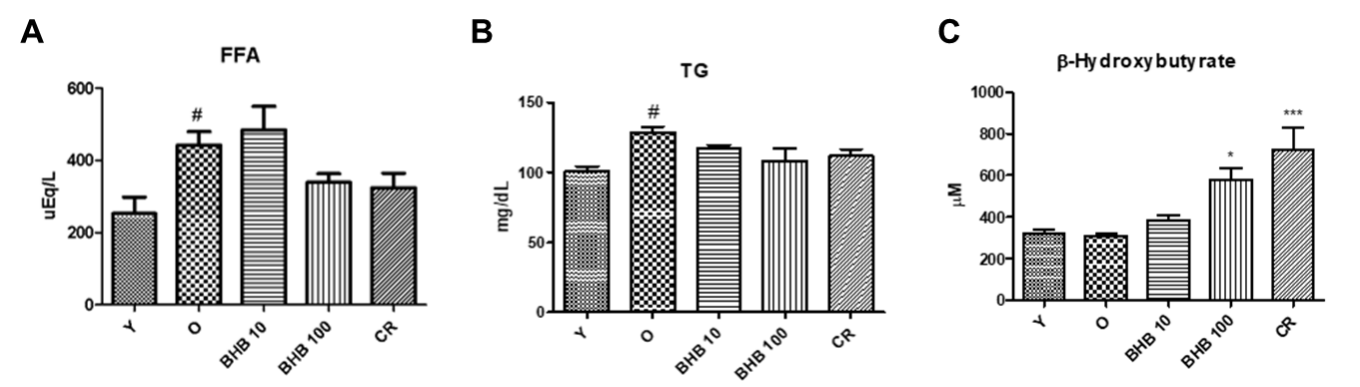
**Changes in lipid and β-hydroxybutyrate levels in the serum**. A series of plasma profiles from BHB-treated or calorie-restricted aged rats are shown. Aged rats (24 months old) were treated with BHB for 30 days (10 or 100 mg/kg/day P.O.) and the respective parameters were compared with young rats (6 months old). BHB was administered to the aged rats (n = 4 each). **(A)** Free fatty acid (FFA), **(B)** triglyceride (TG), and **(C)** β-hydroxybutyrate levels were measured after 30 days of BHB treatment. One-factor ANOVA was used to determine the significant differences. #p < 0.05, ##p < 0.01 vs. young; *p < 0.05, ***p < 0.001 vs. old. Y, young rats (6 months old).

### Statistical analyses

One-way analysis of variance (ANOVA) was used to compare the differences among three or more groups. Differences in the means of the individual groups were assessed by Newman-Keuls *post-hoc* test. Student’s *t*-test was used to analyze the differences between two groups. A p-value of <0.05 was considered significant. All analyses were performed using GraphPad Prism 5 (GraphPad, La Jolla, CA, USA).

**Figure 2.**
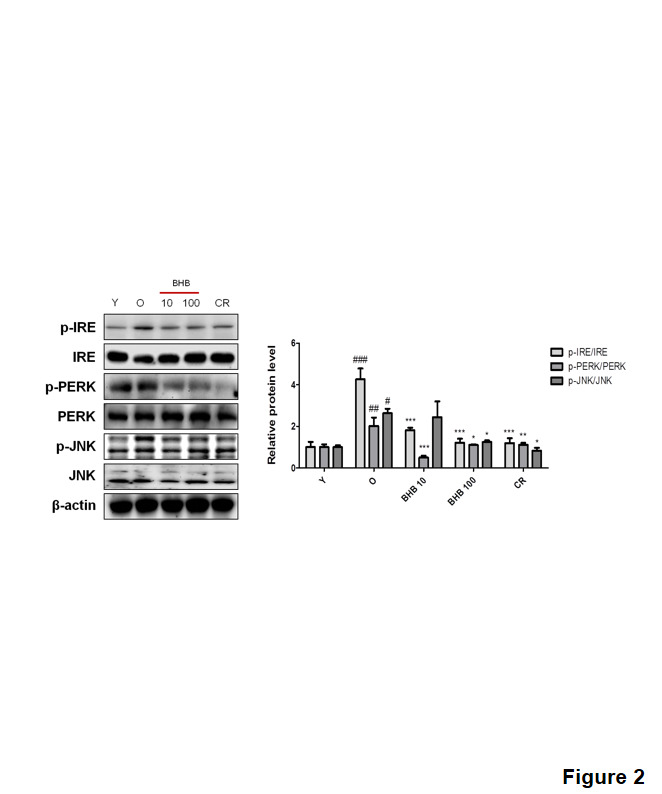
**Effects of BHB on ER stress in the liver of the aged rats**. Western blotting was performed to detect the levels of the ER stress markers (p-PERK and p-IRE), and the downstream signal p-JNK. Western blot results from three independent experiments were quantified by densitometry. One-factor ANOVA was used to determine the significant differences. #p < 0.05, ##p < 0.01, ###p < 0.001 vs. young; *p < 0.05, **p < 0.001, ***p < 0.001 vs. old.

**Figure 3.**
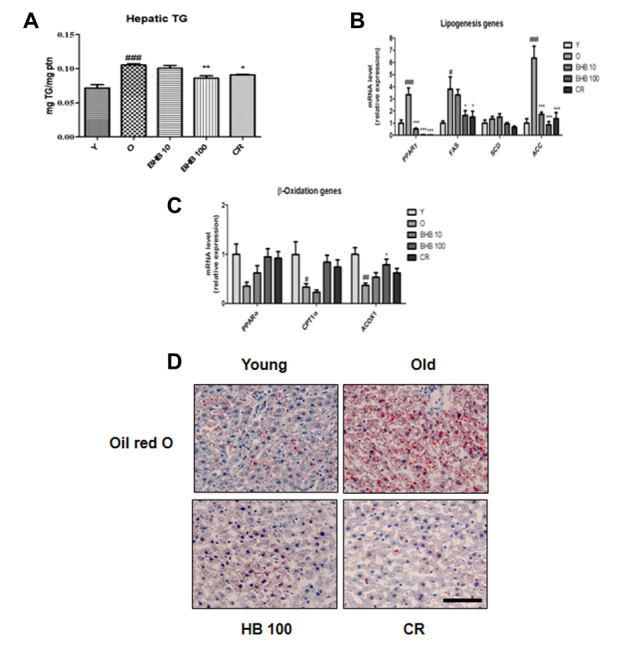
**Changes in lipid accumulation, lipogenic genes, and β-oxidation genes in the livers of BHB-treated aged rats**. **(A)** Hepatic TG contents, **(B)** mRNA expression of lipogenic genes, and **(C)** β-oxidation genes were evaluated by q-PCR. The results were normalized to the expression of a reference gene (GAPDH). One-factor ANOVA was used to determine the significant differences. #p < 0.05, ##p < 0.01, ###p < 0.001 vs. young; *p < 0.05, **p < 0.01, ***p < 0.001 vs. old. **(D)** Changes in lipid accumulation in calorie-restricted (CR) and BHB-treated aged rats. Aged liver tissues were stained with Oil red O to visualize lipid accumulation. Scale bar: 400 μm.

## RESULTS

### Changes in the metabolic parameters during aging

To investigate the physiological alterations, the metabolic parameters in the young, old, CR, and BHB-treated old rats. Serum FFA, TG, and BHB were measured in the 6-month-old, 24-month-old, and BHB-treated 24-month-old rat serum. The Table 2 lists the changes in body weight, food intake, and liver weight during the experimental period. The serum levels of the different parameters were estimated to determine the effects of BHB on the metabolic changes during the aging process (Fig. 1). In the aged rats, FFA and TG levels were significantly elevated. BHB administration induced a reduction in serum FFA and TG levels as compared to the old rats. Similarly, CR induced a reduction in serum FFA, and TG levels as compared to the old rats. The BHB level was not changed in the young and old rats. However, the BHB level in BHB-treated old rats was higher than that of the old rats and was shown to be similar to CR group. These results suggest that BHB altered the metabolic parameters in the old rats, implying that BHB administration may ameliorate lipid accumulation.

**Figure 4.**
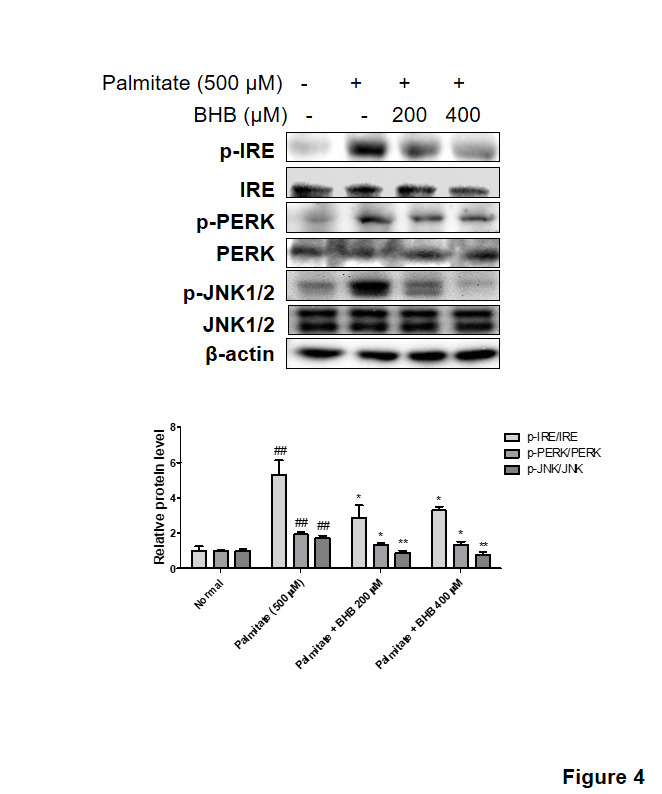
**Effects of BHB on palmitate-induced ER stress**. HepG2 cells incubated with BHB (200 and 400 µM) for 3 h followed by treatment with palmitate (500 µM) for 24 h. Western blotting was performed to detect the levels of the ER stress markers (p-PERK and p-IRE). and the downstream signal p-JNK. Western blot results from three independent experiments were quantified by densitometry. One-factor ANOVA was used to determine the significant differences. ##p < 0.01 vs. normal; *p < 0.05, **p < 0.01 vs. palmitate (500 µM).

### Effects of BHB on ER stress and lipid accumulation

The protein levels of IRE phosphorylation were higher in the old rats than those in the young rats, while CR and BHB administration reduced IRE phosphorylation (Fig. 2). IRE led to JNK phosphorylation in the old rats, whereas BHB-treated old rats showed attenuated protein level of ER stress markers including PERK. To verify the preventive effects of BHB on lipid accumulation, the hepatic TG content was examined. Fig. 3A shows the results of the hepatic TG content. The level of hepatic TG was significantly increased in the old rats as compared to that in the young rats, whereas CR and BHB-treated old rats showed comparatively lower hepatic TG levels. To determine the effects of BHB on lipid metabolism-associated gene expression, qPCR was performed. As shown in Fig. 3B, the expression of lipogenesis-related genes (PPARγ, FAS, SCD, and ACC) was increased in the old rats, whereas that in the CR and BHB-treated old rats was reduced. Moreover, CR and BHB-treated old rats showed comparatively higher expression of β-oxidation-related genes (PPARα, CPT-1α, and ACOX1) than that in the old rats (Fig. 3C). These results suggest that BHB and CR improve hepatic lipid accumulation through down-regulation of lipogenic genes and up-regulation of β-oxidation genes. These results were confirmed by Oil red O staining of the liver tissues. We further detected an overall increase in the number of vacuoles in the liver tubules during the aging process. The CR or BHB-treated groups were found to suppress lipid accumulation (Fig. 3D).

### Effects of BHB on palmitate-induced ER stress and lipid accumulation in HepG2 cells

**Figure 5.**
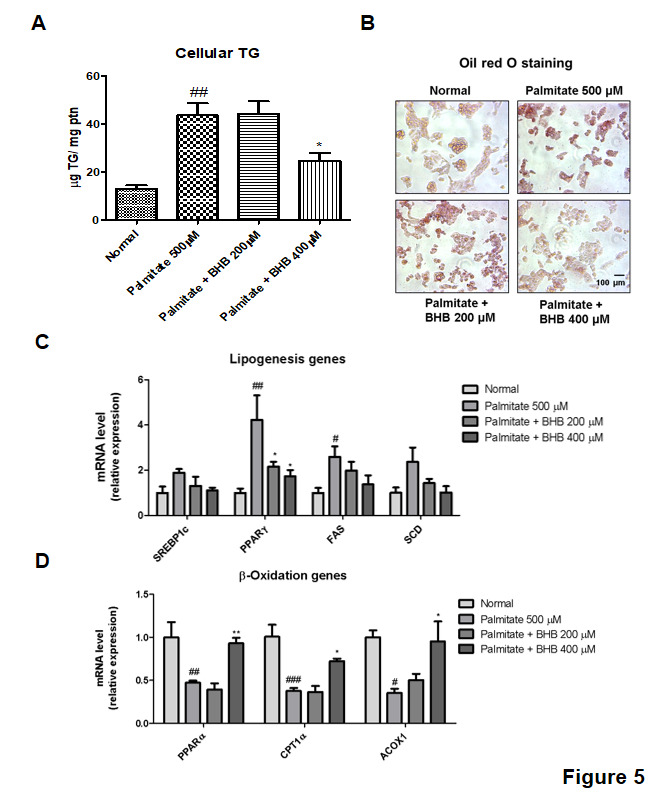
**Changes in lipid accumulation in the BHB-treated HepG2 cells**. **(A)** Cellular TG content and **(B)** Oil red O staining of palmitate-induced HepG2 cells are shown. HepG2 cells were pre-treated with BHB (200 and 400 µM) for 3 h followed by treatment with palmitate (500 µM) for 24 h. Scale bar: 100 µm. mRNA expression of **(C)** lipogenic genes and **(D)** β-oxidation genes were evaluated by q-PCR. The results were normalized to the expression of a reference gene (GAPDH). One-factor ANOVA was used to determine the significant differences. #p < 0.05, ##p < 0.01, ###p < 0.001 vs. normal; *p < 0.05, **p < 0.01 vs. palmitate (500 µM).

Palmitate, which is one of the FFA, alters ER environment, and as a consequence, misfolded proteins are produced. Misfolded protein accumulation in the ER, in turn, may activate UPR [23]. To investigate the modulatory effects of BHB on ER stress, cells were used for Western blotting. As shown in Fig. 4, the protein levels of ER stress markers including p-PERK, p-IRE, and p-JNK were increased in the palmitate-treated HepG2 cells. On the other hand, BHB reduced the levels of ER stress-related proteins. Further to determine whether BHB inhibits lipid accumulation in HepG2 cells, the level of cellular TG was measured. BHB-treated groups reduced cellular TG level as compared to that in the palmitate-treated group (Fig. 5A). Similarly, lipid accumulation was examined by Oil red O staining. As shown in Fig. 5B, lipid droplets were highly accumulated in the palmitate-treated HepG2 cells, whereas BHB-treated HepG2 cells showed reduced lipid droplets. To determine the effects of BHB on lipid metabolism associated gene expression, qPCR was performed. As shown in Fig. 5C, the expression of lipogenesis-related genes (SREBP-1c, PPARγ, FAS, SCD, and ACC) was increased in the palmitate-treated HepG2 cells, whereas that in the BHB-treated HepG2 cells was reduced. Moreover, BHB showed comparatively greater expression of β-oxidation-related genes (PPARα, CPT-1α, and ACOX1) than that in the palmitate-treated HepG2 cells (Fig. 5D). These results suggest that BHB and CR improve hepatic lipid accumulation through down-regulation of lipogenic genes and up-regulation of β-oxidation genes.

### Modulation of GPR109A downstream signaling by BHB in the aged rat liver

**Figure 6.**
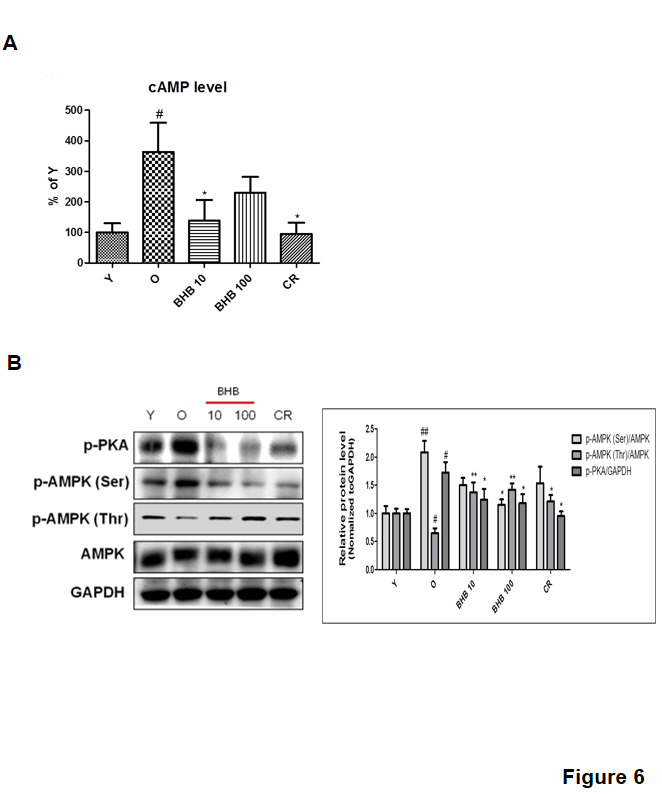
**Changes in AMPK phosphorylation through the GPR109A signaling pathway in BHB-treated rat liver**. **(A)** cAMP level in the liver homogenate. **(B)** Western blotting was performed to detect the levels of p-PKA, p-AMPK (Ser), p-AMPK (Thr), and AMPK in rat liver. Western blot results from three independent experiments were quantified by densitometry. One-factor ANOVA was used to determine the significant differences. #p < 0.05, ##p < 0.01 vs. young; *p < 0.05, **p < 0.01 vs. old.

As shown in Fig. 6A, the cAMP level was significantly increased in the old rats as compared to that in the young rats, whereas the CR and BHB-treated old rats showed comparatively lower levels of cAMP. BHB can reduce the intracellular cAMP concentration by activating the Gαi subunit of GPR109A [16]. To investigate the cAMP-dependent PKA signaling pathway, western blotting was performed. The PKA phosphorylation was lower in the CR and BHB-treated old rats than that in the old rats. BHB-dependent inhibition of PKA phosphorylation led to reduced phosphorylation of AMPK at Ser173 and this reciprocally activated AMPK-Thr172 phosphorylation [17]. Next, we performed an experiment to verify whether BHB inhibits phosphorylation at the Ser173 site in AMPK. It was observed that the induction of AMPK-Thr172 phosphorylation by BHB was associated with a concomitant reduction in AMPK-Ser173 phosphorylation (Fig. 6B). These results suggested that BHB reduced cAMP level leading to a subsequent reduction in PKA phosphorylation and AMPK-Ser173 phosphorylation. Thus, BHB inhibited PKA phosphorylation by reducing the cAMP level in the BHB-treated old rats.

### Modulation of GPR109A signaling by BHB in HepG2 cells

**Figure 7.**
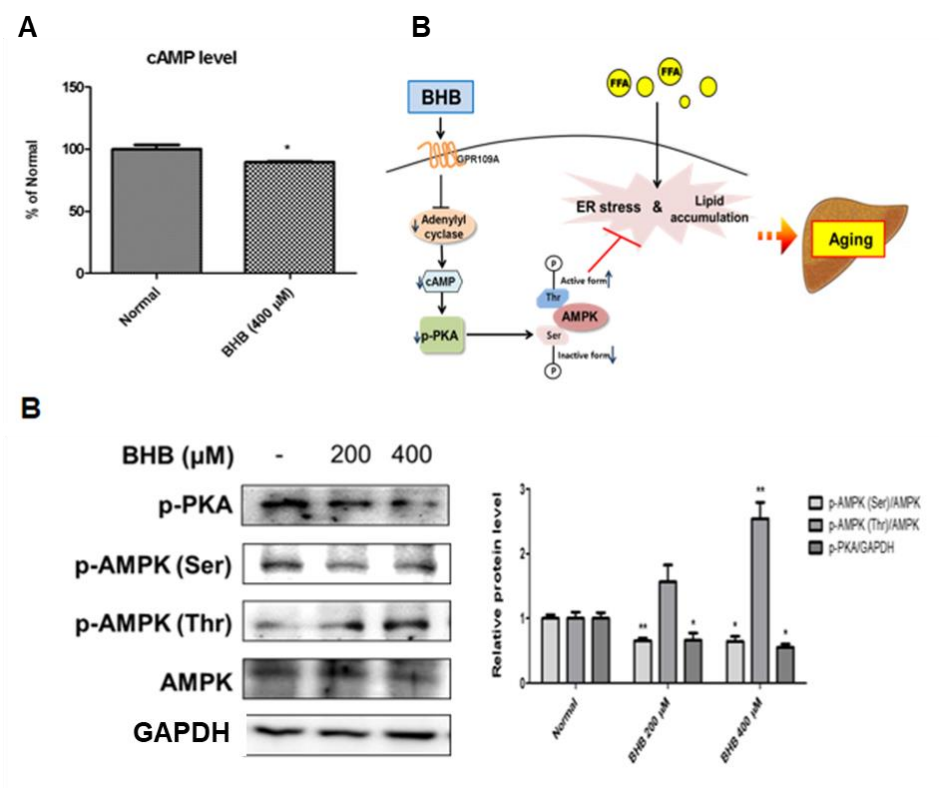
**Effects of BHB on AMPK-Thr172 phosphorylation through the GPR109A signaling pathway in HepG2 cells**. **(A)** Intracellular cAMP was treated with 400 µM BHB for 1 h in HepG2 cells. The results of Student’s *t*-test are shown. *p < 0.005 vs. normal. **(B)** HepG2 cells were incubated with BHB (200 and 400 µM) for 6 h. Western blotting was performed to detect the levels of p-PKA, p-AMPK (Ser), p-AMPK (Thr), and AMPK in HepG2 cells. Western blot results from three independent experiments were quantified by densitometry. One-factor ANOVA was used to determine the significant differences. *p < 0.05, **p < 0.01 vs. normal. **(C)** The possible mechanisms of the protective effects of BHB on hepatic lipid accumulation during aging are shown. Activation of GPR109A inhibited adenylyl cyclase and subsequent reduction in PKA activity by BHB. PKA dephosphorylation reduced AMPK-Ser173 and increased AMPK-Thr172 phosphorylation by BHB. Increased AMPK activity inhibited ER stress and lipid accumulation.

As shown in Fig. 7A, BHB reduced cAMP level in HepG2 cells. To study the cAMP-dependent PKA signaling pathway, western blotting was performed. The PKA phosphorylation was lower in the BHB-treated HepG2 cells than that in the normal group. We next investigated whether BHB reduced phosphorylation at the Ser173 site of AMPK. The induction of AMPK-Thr172 phosphorylation by BHB was associated with a concomitant reduction in phosphorylation of AMPK at Ser173 (Fig. 7B). These results demonstrated that BHB decreased cAMP and cAMP-dependent PKA phosphorylation leading to regulatory AMPK phosphorylation, in which decreased AMPK-Ser173 and increased AMPK-Thr172. Thus, BHB inhibited PKA phosphorylation by reducing the cAMP level in HepG2 cells. This confirmed a reduction in the intracellular cAMP level and a subsequent reduction in the PKA activity. Inhibition of PKA activity blocks AMPK-Ser173 phosphorylation, thereby increasing AMPK-Thr172. These results suggest that BHB reduced intercellular cAMP level leading to the regulation of the GPR109A/AMPK pathway in HepG2 cells.

### BHB regulates ER stress and lipid accumulation through GPR109A/PKA/AMPK in the cells

**Figure 8.**
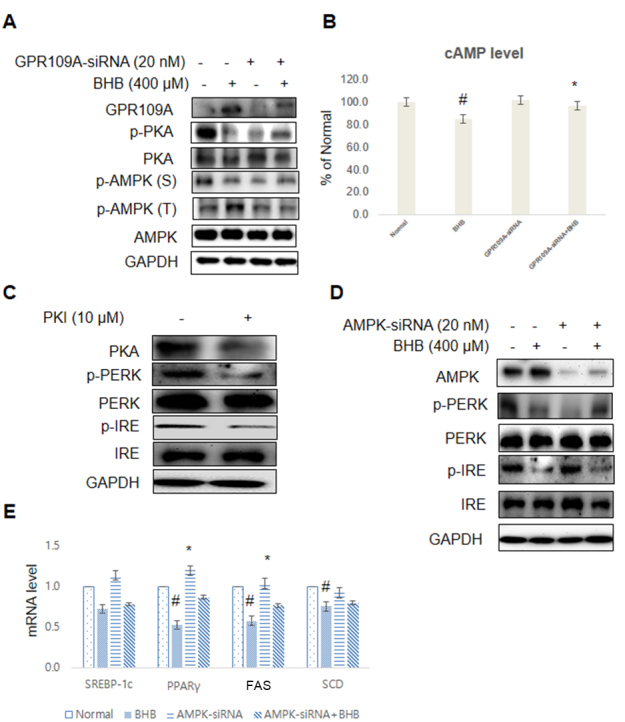
**BHB suppressed ER stress and lipid accumulation through GPR109A/PKA/AMPK in the cells**. **(A)** The cells were treated with the BHB (400 µM) for 4 h. The cells pre-incubated with GPR109A-siRNA (20 nM) for 44 h were subjected to western blotting analysis using GAPDH as control. GPR109A, p-PKA, PKA, p-AMPK (Ser), p-AMPK (Thr), and AMPK levels were assessed. **(B)** The cAMP level measured after stimulation with BHB in the absence (-) or presence (+) of GPR109A-siRNA in the liver. The results of Student’s *t*-test are shown. ^#^p < 0.05 vs. normal; ^*^p < 0.05 vs. GPR109A-siRNA treated cells. **(C)** HepG2 cells were transfected and pre-incubated with PKI (10 µM) for 1 h. The cells were analyzed by Western blotting using PKA, p-PERK, PERK, p-IRE, IRE, and GAPDH antibodies. **(D)** The cells were treated with the BHB (400 µM) for 4 h. The cells pre-incubated with AMPK-siRNA (20 nM) for 44 h were subjected to western blotting analysis using GAPDH as control. AMPK and ER stress markers (p-PERK, PERK, p-IRE, and IRE) were assessed using cytosolic proteins from HepG2 cells. **(E)** The cells incubated with BHB (400 µM) for 4 h. The cells pre-transfected with AMPK-siRNA (20 nM) for 44 h were subjected to qPCR analysis using actin as a control. The mRNA expression of the lipogenic genes (SREBP-1c, PPAR, FAS, and SCD) was assessed. The results were normalized with respect to the actin level. ^#^p < 0.05 vs. Normal; ^*^p < 0.05 vs. AMPK-siRNA group.

To further illustrate the importance of BHB, we used the siRNA-mediated gene approach to knock down GPR109A expression in HepG2 cells by treating the cells with GPR109A-siRNA followed by BHB treatment at 400 μM. As shown in Fig. 8A, BHB suppressed PKA and AMPK-Ser phosphorylation and reduced phosphorylated AMPK-Thr levels in the GPR109A-siRNA-treated HepG2 cells. However, BHB treatment reduced cAMP level. Otherwise, no change in cAMP level in GPR109A-siRNA was observed (Fig. 8B). We examined the effect of ER stress genes on the PKA inhibitor, PKI. In these experiments, the liver cells were treated with PKI for 1 h. As shown in Fig. 8C, the ER stress genes were suppressed by PKI. We also investigated whether BHB treatment induced AMPK expression in the absence or presence of AMPK (Fig. 8D). BHB was found to induce AMPK expression. In addition, BHB suppressed mRNA expression of the lipogenesis genes, but AMPK-siRNA treatment did not affect the mRNA level of the lipogenic genes (Fig. 8E).

## DISCUSSION

Aging is one of the most critical risk factors for the onset and progression of age-related disease including obesity, metabolic syndrome, diabetes mellitus, hypertension, and atherosclerosis. Hepatic lipid accumulation is representative of the pathological phenomenon of age-related liver diseases, such as hepatitis, cirrhosis, and cancer. Fatty liver disease is considered to be the consequence of metabolic diseases accompanied by increased serum FFA, TG, and LDL levels, as well as decreased HDL level. The lipotoxic environment of non-alcoholic fatty liver disease (NAFLD) is due to a surplus of lipids that directly influence ER homeostasis and ER stress activation [24].

ER stress is known as UPR, which is triggered by the accumulation of unfolded proteins in the ER lumen. This response is important for the restoration of normal ER function. Prolonged ER stress causes cell death, prevents translation of synthesized protein, and increases the production of ER chaperones, such as glucose-regulated protein (GRP) 78 and 94. ER stress is also considered as the critical component in the development of insulin resistance and lipid accumulation. Three protein are well known to sense ER stress and activate UPR: PERK, IRE-1, and ATF6 [25]. Recently, it has been reported that lipid accumulation and ER stress were ameliorated by CR and intermittent fasting dietary intervention as indicated by a decrease in the hepatic TG level under these conditions [26, 27].

The above studies provide evidence suggesting that BHB could be used as a potential CR mimetic, which has beneficial anti-lipogenic effects in the liver as demonstrated *in vivo* and *in vitro*. CR prolongs the life span and is known to modulate the onset and progression of age-related diseases in the non-vertebrates and rodents. The beneficial effects of CR have been well-established through experiments and there is accumulating evidence on its anti-aging effects. Previous studies have shown that CR suppresses systemic inflammation and may modulate fatty liver disease [27, 28]. Moreover, CR changes hepatic lipid droplet proteome and diacylglycerol species and also prevents diabetes in mice [26]. BHB is a metabolic product that is released into the blood circulation mostly from the liver and is utilized in the extrahepatic tissues in the fasting state. In humans, basal serum levels of BHB are normally maintained in a low micromolar concentration range, but the level begins to rise to a few hundred micromoles after 12-16 h of fasting, reaching 1-2 mM after 2 days of fasting [29]. Furthermore, a prolonged CR condition leads to an increased BHB level in rats [30].

Consistent with the results of previous studies, serum BHB level was notably increased in the present study in rats conditioned by CR similar to that of rats administered BHB at a high dose. The serum levels of TG and FFA were diminished in both CR and BHB-treated aged rats (Fig. 1). A possibility is that BHB exerts inhibitory effects on hepatic lipid accumulation. Based on the hepatic TG contents analysis, BHB administration in a high dose showed reduced hepatic TG levels and increased expression of CPT-1, a key regulatory enzyme of β-oxidation, thus, up-regulating β-oxidation genes, such as ACOX1 (Fig. 3C). In line with such speculation, the aging process was found to increase hepatic lipid accumulation, whereas this phenomenon was compromised in the BHB-treated old rats (Fig. 3).

It has been reported that an adequate concentration of BHB during starvation could reach to bind and activate GPR109A as its receptor. GPR109A activation through BHB inhibits adipocyte lipolysis [14, 31]. Therefore, it has been suggested that BHB acts as a metabolite and aids in survival during starvation. It regulates self-production, prevents ketoacidosis, and regulates lipid utilization. In GPR109A knockout mice, increased body weight, hepatic steatosis, and serum biomarkers of liver injury were observed [15]. The present study focused on the regulatory mechanism of BHB signaling through GPR109A. GPR109A interacts and couples to a Gαi subunit protein and inhibits AC activity.

GPR109A is known to signal and inhibits PKA activity [32]. PKA activation leads to AMPK-Ser173 phosphorylation of the inactive form, as well as inhibition of AMPK-Thr172 phosphorylation. Thus, this observation supports that BHB acts as a ligand of GPR109A in the liver. A central feature in the proposed model involves PKA-dependent AMPK-Ser173 to inhibit AMPK-Thr172 phosphorylation. In regard to this reciprocal mechanism, AMPK-Ser173 phosphorylation was found to inhibit AMPK-Thr172 phosphorylation by PKA [17]. In line with this report, increased AMPK-Ser173 phosphorylation reciprocally reduced AMPK-Thr172 phosphorylation leading to the down-regulation of AMPK activity [18]. AMPK activation via AMPK-Thr172 phosphorylation prevented ER stress response and hepatic lipid accumulation [19]. Inhibition of lipid accumulation is mediated by BHB-dependent AMPK-Thr172 phosphorylation that occurs when AMPK-Ser173 phosphorylation is suppressed. Therefore, these data suggest that GPR109A signaling regulates the reciprocal activation mechanism between AMPK-Thr172 and AMPK-Ser173 phosphorylation that contributes to the abilities of BHB to improve hepatic lipid accumulation (Fig. 7).

AMPK has been shown to play a critical role in controlling the systemic energy balance and metabolism [33]. Furthermore, AMPK may be a physiological regulator that maintains ER homeostasis [34] and protects against hepatic steatosis when mice are fed on a high-fructose diet [35]. However, this study has certain limitations as we primarily explored the acute effect of BHB. We administered BHB only for 30 days, which is considered a rather short induction period, and the treatment period could have been prolonged further to define the molecular mechanisms representing the anti-lipid accumulation effect *in vivo* and *in vitro*. In this study, we explored the beneficial effects of BHB on liver steatosis, suggesting that BHB supplementation may lead to the restoration of liver functions in the aging process. BHB regulates the activation of GPR109A and leads to a reduction in cAMP level in the liver, resulting in the inhibition of PKA phosphorylation. PKA inhibition inhibits AMPK phosphorylation at the Ser173 site, which is an inactive form and stimulates AMPK-Thr172 phosphorylation. Therefore, BHB-mediated AMPK activation attenuates ER stress and lipid accumulation in the aged liver (Fig. 7C). Our results provide significant insights into the cellular and molecular basis of GPR109A/PKA/AMPK, thereby proposing this novel candidate target for the treatment of altered lipogenesis.

In summary, BHB administration could improve age-related ER stress and lipid accumulation through stimulation of the GPR109A pathway. This mechanism provides a unifying model to account for the therapeutic effects of BHB through the GPR109A/AMPK signaling. We conclude that BHB suppresses aging-related ER stress and lipid accumulation as a CRmimetic via GPR109A/ AMPK in aged liver. Therefore, activation of AMPK by BHB through the GPR109A pathway in the liver has the potential to improve deranged metabolic functions in fatty liver.
